# Social Support of Organ Donor Families in China: A Quantitative and Qualitative Study

**DOI:** 10.3389/fpubh.2021.746126

**Published:** 2021-11-17

**Authors:** Aijing Luo, Haiyan He, Zehua Xu, Xuantong Deng, Wenzhao Xie

**Affiliations:** ^1^The Third Xiangya Hospital of Central South University, Changsha, China; ^2^Key Laboratory of Medical Information Research (Central South University), College of Hunan Province, Changsha, China; ^3^School of Life Sciences, Central South University, Changsha, China; ^4^Public Health College of Central South University, Changsha, China

**Keywords:** organ donation, donor families, social support, social support needs, coping styles

## Abstract

**Background:** Donor families experienced a difficult time during and after the process of organ donation. There is a necessity to understand the support they received and what they need to help them get through a painful time. This study aimed to investigate the social support level and social support needs of the donor families in China.

**Methods:** A cross-sectional study was conducted among 102 donor families using a questionnaire to investigate their demographics and social support level. To further understand their social support needs, in-depth interviews were conducted among 9 donor families.

**Results:** Findings of the study showed that (1) Most of the family members (74, 72.6%) lacked social support, and only a small number of families (28, 27.5%) received sufficient social support (2). The coping style had an impact on the overall social support level (*P* = 0.014) (3). There was a lack of emotional support, information support and material support toward the donor's family members. Both emotional support and material support are significantly needed.

**Conclusions:** The overall social support level remained insufficient and the utilization degree of social support was low. Organ donor families are in desperate need of material and emotional support. The level of social support is largely influenced by the donor familie's coping style. Compared with a negative coping style, donor families who adopted a positive coping style acquire more social support.

## Introduction

Organ donor shortage has become a crisis due to the significant mismatch between the increasing demand for transplantation and the limited availability of donors in the world. According to statistics, 17 patients in the USA died every day while waiting for donor organs (1). The demand for donor organs in China has been increasing by 12% each year (2). Since January 1, 2015, China has stopped the use of organs of death row prisoners for transplantation, voluntary organ donation after the death of the citizens has become the only channel for organ transplantation (3). China has become the second-largest country of organ transplantation with the number of organ transplantation hospitals amount to 170 all over the country (4). According to the Human Organ Donation Management Center of China, there were a total of 36,432 organ donation cases, and 10,8610 large organs of various types have been donated as of December 2021, ranking top in the number of organ donations worldwide (5). A voluntary organ donation is an altruistic act of love. Organ donors are expected to be respected in every country and be cared for. However, relatives of organ donors are the ones who are most likely to experience psychological disorders and stress during the process of organ donation (6, 7). Donor families experienced extreme emotions, psychological dynamics, and anticipatory grief (8, 9). A study showed that even after organ donation, donor families would still suffer bereavement, post-traumatic stress disorder, and depression (10).

Social support is regarded as a moderator of life stress (11). No guideline has clearly defined social support, but it is generally considered as services, care, or encouragement provided by the members of social networks (usually spouses, partners, family and friends) (12). Previous social support studies focused on specific diseases, such as diabetes, depression, AIDS, cancer and specific groups. Deshira D. Wallace highlighted the need to assess and leverage distinct types and sources of social support at different stages of the diabetes experience (13). Significant associations were found between diabetes type and social network, social support and health behavior (14). Besides, both diabetes-related medical symptoms and social support independently contributed to depression (15), depression also contributed to lower social support (16). Studies showed HIV risk behaviors increase with mental health needs and decrease with the level of social support (17). People who live with HIV/AIDS prefer to seek social support online (18). Social support is significantly needed among people who suffered from cancer (19–22). Besides, elderly people's quality of life was also influenced by social support (23, 24). Previous studies declared that increased social support should be given to pregnant women (25–27) and LGBT youth (28–30). However, little attention was paid to the social support of the organ donor's families. Relevant research revealed that the family members of organ donors lacked social and emotional support during the decision-making process (31). Moreover, adequate social support can avoid family conflict toward living kidney donor transplantation (32).

Currently, the lack of social support represents contraindication for organ transplantation and organ donation (33, 34). The number of organ donors has become short mainly due to the traditional cultural factors and limited relevant incentive policies in China. Confucian filial piety demands that the body should be kept intact and respected. Although cremation is carried out in many places of China, people prefer that the body remain intact (35). Therefore, the degree of social support received by donor families is relatively low in China. However, most of the organ donors and their families belong to socially vulnerable groups, among which there are some orphans and some families who lost their only child. Zhao Baige, the executive vice president of the Red Cross Society of China, said that more than 90% of the families were involved in the problem of applying for assistance in difficult times (36). Understanding the social support of donor families can enable coordinators to help families alleviate psychological distress (8). Identifying the specific support needs of family members is critical in helping them to cope with this situation (37).

Therefore, We attempted to explore the social support level and social support needs of the donor families. Taking Hunan Province as an example, this paper conducted a cross-sectional survey on the social support of organ donor families with quantitative and qualitative research methods. This study was helpful to provide a reference for the government departments in formulating relevant policies and targeting intervention measures.

## Methods

### Research Participants

Based on the principle of voluntary consent and convenient sampling method, a questionnaire and qualitative survey were conducted on the immediate family members of organ donors, including but not limited to spouses, parents, grandparents and children. The study was carried out in the Hunan Province from April to August 2017. The inclusion criteria were as follows: ① direct family members of organ donors; ② good communication and reading ability; and ③ voluntary participation.

### Research Tool

① According to the purpose of this study, a General Information Questionnaire including 9 items, such as gender, age, marital status, education level, health status, occupation, income, residence location and kinship with organ donors was compiled.
② Simplified Coping Style Questionnaire, which has good reliability and validity, was used to analyze psychological pressure, anxiety, social avoidance and distress of the family members of the donors. The Cronbach's α coefficient was 0.80, and the validity kmo was 0.714. It consisted of a positive coping style and a negative coping style. The research results showed a significant relationship between an individual's coping style and mental health.
③ Social support rating scale (SSR) consisted of 10 items, including three dimensions: subjective support, objective support and support utilization. The scale has good reliability and validity, with test-retest reliability of 0.92, Cronbach's α coefficient of 0.89–0.94, and validity coefficient of 0.724–0.835.
④ Semi-structured interviews were conducted to further analyze the social support needs of donor families. Interview Outline for the Social Support Level and Needs of the Organ Donors Families in Hunan Province were compiled by the researchers (see Table 1), which mainly focused on emotional support needs, information support needs, and material support needs. The research group followed the principle of informed consent and tried to improve the heterogeneity of interviewees, such as the relationship with donors, gender, age, family background and so on.

**Table 1:** The interview outline for the social support level and needs of the organ donors families in hunan province.

1. How did you get information about organ donation?
2. As a family member of the donor, have you ever been leaked personal information? What measures do you want the government, the Red Cross, hospitals or other official institutions to do in terms of information protection?
3. Do you want to know about the recipient's physical condition and living condition after surgery?
4. Did you consider material support when making the donation decision? Such as financial aid? Have you and your family received material support?
5. Do you regret agreeing to donate your relative's organs? Why?
6. Do you need psychological comfort and support? Have you ever received psychological support? Such as the care of relatives and friends, or professional psychological counseling?

### Data Collection and Processing

#### Questionnaire Distribution

Questionnaires were issued after the informed consent forms were signed by the participants. The questionnaires were distributed and collected on the spot and filled in anonymously. From April to August 2017, we conducted an on-site questionnaire survey in the memorial activity held by the Red Cross Society of Hunan Province held for organ donors. At the same time, the organ donation coordinator was entrusted to send questionnaires to the donor's home. A total of 112 questionnaires were collected, of which 102 were valid and the effective rate was 91%. SPSS18.0 was used for statistical analysis. The analysis methods used included frequency, percentage and chi-square test, and the significance level was α = 0.05.

#### Semi-structured Interview

From April to May 2017, nine immediate relatives of organ donors in Hunan Province were successfully contacted to participate in the interview, and the interview time was limited to 45 min to 1 h. During the interview, the behavior and reaction of the interviewees were observed and recorded. The interview materials were co-coded by two researchers. To protect the privacy of participants, the interviewees are numbered and presented in the form of “D1, D2, D3, D4, D5, D6, D7, D8, and D9.”

### Ethics

Approval for the study was obtained from the Institutional Ethics Committee of the Third Xiangya Hospital, Central South University.

## Results

### Demographic Data of Respondents

The general demographic data of the respondents are shown in Table 2. There were 48 males and 54 females who participated in the survey, with an average age of 42.2 ± 12.7 years, ranging from 13 to 80 years old. Of the 102 participants, 73.5% were married, 54.9% were junior high school degrees or below. Forty-eight participants had a general health condition. The occupation of most of these was peasants. The average monthly income of 46 participants was <1,500 yuan and most were rural residents.

**Table 2:** General demographic data of respondents (*N* = 102).

**Variables**	**Frequency**	**Percentage (%)**
**Gender**		
Male	48	47.1
Female	54	52.9
**Age (year)**		
<25	9	8.8
25~35	23	22.6
>35	70	68.6
**Marriage status**		
Unmarried	12	11.8
Married	75	73.5
Divorced	4	3.9
Widowed	10	9.8
Other	1	1
**Educational level**		
Junior high school or below	56	54.9
Senior high school or junior college	39	38.2
Bachelor degree or above	7	6.9
**Health condition**		
Bad	21	20.6
General	48	47.1
Good	33	32.4
**Occupation**		
Government staff, Business personnel, or technical staff	20	19.6
Peasant	41	40.2
Freelance	17	16.7
Other	24	23.5
**Average monthly income (RMB)**		
<1,500	46	45.1
1,500–5,000	41	40.2
>5,000	15	14.7
**Registered residence**		
City	23	22.6
Countryside	79	77.5
**Relationship with donor**		
Spouse	7	6.9
Parents	47	46.1
Children	15	14.7
Brothers and sisters	22	21.6
Grandfather	1	1
Grandmother	1	1
Grandchildren	1	1
Other	8	7.8

### Results of Simple Coping Style Questionnaire

The coping style of organ donor's family members showed that the coping tendency value ranged from −2.06 to 2.80, with an average of 0.0008 ± 1.04 (see Table 3). Among these, 49 had a positive coping style when the coping tendency value was >0, and 53 had a negative coping style.

**Table 3:** Simple coping style questionnaire score.

	**Variables (Score = S)**	**Frequency**	**Percentage (%)**
Coping tendency value	S > 0	49	48
	S <0	53	52
Highest score	S = 2.80		
Lowest score	S = −2.06		
Positive score	S = 1.65 ± 0.53		
Negative score	S = 1.36 ± 0.55		
Average score	S = 0.0008 ± 1.04		

### Analysis of the Social Support of the Family Members of Organ Donors

#### Overall Level of Social Support

The overall level of social support was reflected by the overall scores of subjective support, objective support and social support utilization. According to the scores of the social support rating scale, <20 points accounted for 0.98%, 73 (71.57%) had scores between 20 and 40, and 28 (27.45%) had scores higher than 40. This suggested that most of the family members received limited social support, and only a small number of families received sufficient social support.

#### Objective Support

Objective support of organ donors families is shown in Table 4. In the last year, 73.53% of the family members mainly lived with their families. The financial support and psychological comfort that they had received were mainly from spouses and relatives. Only a few of these have received official or unofficial support. These results showed that the objective support source of organ donor families was limited, and the financial support and practical help were mainly obtained from the family members.

**Table 4:** Objective support.

**Entry**	**Variable**	**Frequency**	**Percentage (%)**
Q2. In the past year, you	Were away from your family and lived in a single room	12	11.76
	Lived with strangers most of the time	11	10.78
	Lived with classmates, colleagues or friends	4	3.92
	Lived with family	75	73.53
	No source	0	0
Q6. In the past, when you were in a difficult situation, the sources of financial support and help to solve practical problems were from	Spouse	43	42.16
	Other family members	37	36.27
	Relatives	52	50.98
	Colleagues	16	15.69
	Work unit	9	8.82
	Official or semi-official organizations such as party and trade unions	3	2.94
	Non-governmental organizations such as religions and social organizations	0	0
	Other	2	1.96
	No source	0	0
Q7. In the past, when you encountered an emergency, the sources of comfort and care you received were from	Spouse	55	53.92
	Other family members	43	42.16
	Relatives	67	65.69
	Colleagues	33	32.35
	Work unit	8	7.84
	Official or semi-official organizations such as party and trade unions	4	3.92
	Non-governmental organizations such as religions and social organizations	2	1.96
	Other	1	0.98

*Q6 and Q7 are multiple-choice questions, so the sum of frequencies exceeds 100%*.

#### Subjective Support

The results of subjective support of the donor families are shown in Table 5. Nearly half of the families received support from 1–2 friends, while 16.67% of the families didn't get help from friends. Some families received concern from their neighbors and colleagues. Nearly half of the organ donors got full support from husband, wife (lover), and parents.

**Table 5:** Subjective support.

**Entry**	**Variables**		**Frequency**	**Percentage (%)**
Q1. How many close friends do you have that can get support and help	None		17	16.67
	1–2		42	41.18
	3–5		29	28.43
	6 or more		14	13.73
Q3. You and your neighbors	Never care about each other, just nodding acquaintances		17	16.67
	A little concerned when encountering difficulties		21	20.59
	Some neighbors care about you		24	23.53
	Most of the neighbors care about you		40	39.22
Q4. You and your colleagues	Never care about each other, just nodding acquaintances		14	13.73
	A little concerned when encountering difficulties		21	20.59
	Some neighbors care about you		29	28.43
	Most of the neighbors care about you		38	37.25
Q5. Support and care from family members	**None**	**Little**	**General**	**Full support**
Couples (Lover)	14 (13.73)	9 (8.82)	20 (19.61)	59 (57.84)
Parents	18 (17.65)	7 (6.86)	27 (26.47)	50 (49.02)
Children	30 (29.41)	10 (9.8)	23 (22.55)	39 (38.24)
Siblings	10 (9.80)	13 (12.75)	42 (41.18)	37 (36.27)
Other members (such as sister-in-law)	26 (25.49)	25 (24.51)	29 (28.43)	22 (21.57)

#### Utilization of Social Support

The utilization of social support by the families of organ donors is shown in Table 6. Of the investigated family members, 21.57% chose never to tell anyone when they get into trouble. Nearly half of them complained to 1–2 people they were very close to, and most of these were their families. More than half of the donors did not accept or rarely asked for help when they had troubles. A quarter of the participants never participated in group activities, half of them had occasionally participated in group activities. These results suggested that the utilization of social support of organ donor's families remained low.

**Table 6:** Utilization of social support.

**Entry**	**Variables**	**Frequency**	**Percentage (%)**
Q8. Who will you talk to when you are in trouble	Never tell anyone	22	21.57
	Only tell 1–2 people who are very close	47	46.08
	If a friend asks, you will say	18	17.65
	Actively tell your own troubles to get support and understanding	15	14.71
Q9. Who will you ask for help when you are in trouble	Just rely on yourself, don't accept help from others	24	23.53
	Rarely ask others for help	40	39.22
	Sometimes ask someone for help	21	20.59
	When you are in trouble, often ask your family, relatives and friends for help	17	16.67
Q10. For groups (such as party organizations, religious organizations, trade unions, student unions, etc.,) to organize activities, you	Never participate	25	24.51
	Attend occasionally	51	50
	Participate frequently	15	14.71
	Take the initiative and participate in active activities	11	10.78

#### Influencing Factors of Social Support of Organ Donor Families

Taking the total score of social support level as the dependent variable and demographic characteristics and coping style scores of respondents as independent variables, chi-square test was conducted. The results are shown in Table 7. The coping style had an impact on the level of social support, and the difference was statistically significant (*P* = 0.014). The demographic characteristics of family members indicated that no significant differences in the score of social support level (*P* > 0.05).

**Table 7:** Analysis on influencing factors of social support level of organ donor's family members.

**Variables**	**Social support**		**c2**	**p**
	**Lack**	**Adequate**		
**Gender**				
Male	33 (68.7)	15 (31.3)	0.657	0.418
Female	41 (75.9)	13 (24.1)		
**Age (year)**				
<25	8 (88.9)	1 (11.1)	1.831	0.4
25–35	15 (65.2)	8 (34.8)		
>35	51 (72.9)	19 (27.1)		
**Marriage status**				
Unmarried	11 (91.7)	1 (8.3)	10.63	0.031^*^
Married	48 (64.0)	27 (36.0)		
Divorced	4 (100.0)	0 (0.0)		
Widowed	10 (100.0)	0 (0.0)		
Other	1 (100.0)	0 (0.0)		
**Educational level**				
Junior high school or below	41 (73.2)	15 (26.8)	3.486	0.175
Senior high school or junior college	30 (76.9)	9 (23.1)		
Bachelor degree or above	3 (42.9)	4 (57.1)		
**Health condition**				
Bad	18 (85.7)	3 (14.3)	3.147	0.207
General	35 (72.9)	13 (27.1)		
Good	21 (63.6)	12 (36.4)		
**Occupation**				
Other	45 (73.8)	16 (26.2)	0.114	0.736
Peasant	29 (70.7)	12 (29.3)		
**Average monthly income (RMB)**				
<1,500	34 (73.9)	12 (26.1)	3.447	0.179
1,500–5,000	32 (78.1)	9 (21.9)		
>5,000	8 (53.3)	7 (46.7)		
**Registered residence**				
City	19 (82.6)	4 (17.4)	1.509	0.219
Countryside	55 (69.6)	24 (30.4)		
**Anxiety**				
No	26 (70.3)	11 (29.7)	0.151	0.697
Yes	48 (73.9)	17 (26.2)		
**Attitude**				
Negative	44 (83.0)	9 (17.0)	6.073	0.014^*^
Positive	30 (61.2)	19 (38.8)		

* *statistically significant*.

To further clarify the role and trend of social support of organ donor's families, logistic regression analysis was conducted. The odds ratio (OR) and its 95% confidence interval (CI) were used to estimate the protective as well as risk factors. These results showed that compared to negative coping style families, the families with positive coping style act as protective factors of social support (or = 0.323, 95% CI: 0.129–0.810), and this meant that the family members with positive coping style received more social support.

### Interview Results

A total of 9 interviewees officially participated in this study. The general demographic data of the interviewees are shown in Table 8.

**Table 8:** General demographic data of interviewees.

**Number**	**Relationship**	**Interviewees**			**Donors**			
		**Age (year)**	**Location**	**Education level**	**Age (year)**	**Gender**	**Cause of death**	**Donate time**
D1	Father	47	Countryside	Illiteracy	24	Male	Suicide	20/12/2016
D2	Elder sister	52	Countryside	Illiteracy	36	Male	Car accident	25/08/2012
D3	Father	67	City	Senior high school	33	Male	Illness	02/04/2014
D4	Father	54	Countryside	Junior high school	25	Male	Cerebroma	27/02/2016
D5	Father	49	City	Undergraduate	21	Male	Cerebroma	18/12/2012
D6	Father	47	Countryside	Junior high school	19	Male	Car accident	28/11/2013
D7	Elder sister	50	City	Junior high school	43	Male	Encephalorrhagia	01/09/2014
D8	Father	52	City	Junior college	15	Female	Glycogen storage syndrome	08/10/2010
D9	Mother	34	Countryside	Junior high school	7	Female	Cerebroma	23/12/2014

#### Analysis of Social Support Needs of the Family Members of Organ Donors

##### Emotional Support Needs

In-depth interviews were conducted with the nine donor families. The interviews showed that all families hoped that the government would establish a psychological counseling agency for the donor families to relieve psychological pressure. Losing-single-child family (D1 and D3) showed stronger emotional support needs. Six participants were affected by public pressure and hoped to gain understanding and respect from their families, friends, and surrounding people.

D1: *I hope relatives, friends and coordinators come to see me. The government should establish psychological counseling institutions so that we can communicate with professional people more comfortably*.
D3: *I hope my friends, relatives and Red Cross personnel would visit and comfort me. The government and professional institutions should help the families out of the psychological dilemma*.

##### Information Support Requirements

The study showed that all participants wanted to know the health status of the recipients after the operation. Three participants (D2, D3, and D4) mentioned that government departments and the public should pay attention to donor families, and the donation behavior needed to be recognized by society. Four participants (D2, D3, D6, and D8) proposed that the relevant departments and staff should protect the personal information of the donor's families.

D3: *I want to know the process and medical knowledge of organ donation. We should vigorously publicize organ donation. The Red Cross Society and the hospital should protect the personal information of donor families. The act of donation can be publicized, but it is not specific to one person. My biggest wish is to know whether the recipients are in good health*.
D4: *We hope to have sound regulations and policies on organ donation. The main reasons for the rumors and incomprehension are that the superior departments do not pay attention to organ donation and the public publicity is not in place*.

##### Material Support Needs

Several families of donors indicated they needed financial assistance. Participants D1, D3, and D6 showed that the families who lost their only child have a greater demand for pension and employment security. Especially for the families who have lost their main labor force. They need the help of government departments and community organizations in the economy and employment. Besides, the donor families stressed that they hoped the Red Cross would carry out memorial activities for organ donors on Tomb-Sweeping Day in memory of the donors.

D1: *I hope to reduce the hospitalization expenses, funeral expenses and increase the endowment insurance*.
D3: *We hope that government departments will formulate policies to provide old-age security and set up nursing homes for families who have lost their families*.

#### Social Support Analysis of Organ Donor's Family Members

##### Emotional Support

The main forms of emotional support for the donor families include visiting relatives and friends, going out for relaxation, telephone consolation, organizing collective activities and green channels provided by the hospital during the treatment. We noticed that two participants (D1 and D3) who lost their only child received more care and more forms of emotional support, such as chatting with relatives and friends, and the Red Cross's company during the donor's hospitalization.

D2: *The coordinator, the Red Cross Society and township cadres, my relatives, friends and neighbors have visited my family. There will be telephone greetings every year when sweeping graves*.
D3: *My friends and colleagues showed concern for me. I am very grateful to the Red Cross for their company in the hospital for more than 20 days*.

##### Information Support

According to the interviews, four participants obtained organ donation information through the hospital and Red Cross staff. Among them, participants D2 and D7 saw the media publicity and actively contacted the media or Red Cross Society to express their willingness to donate. D5 and D6 said that organ donation coordinator's publicity on organ donation knowledge is essential. It is found that donors and their families themselves, as disseminators of information, bring good publicity effect to relatives, friends and even the public. Besides, lacking publicity on the donation, pressure from public negative opinions and the low recognition of donation behavior are the main reasons why the donor's family members are not willing to disclose their identities.

##### Material Support

Seven participants received financial assistance from the Red Cross Society, and two participants obtained the government's minimum living allowance. Six participants said that they did not consider financial compensation and emphasized voluntary organ donation. Among them, D3 said that the medical support provided by the Red Cross Society and the hospital was also an important factor in making the donation decision at that time.

## Discussion

### Analysis of Social Support Needs of Organ Donor Families

In terms of emotional needs, family members hope to get emotional support from their relatives. Emotional support and help from relatives are essential (6). Timely intervention on psychological changes and bad emotions of donor families at different stages can ease and improve their mood (38). This study showed that donor families received less emotional care from society, and the psychological pressure of some family members comes from the pressure of traditional beliefs and the public. Therefore, the families of organ donors strongly hope to be understood by the public.

In terms of information needs, donor' families hope to know more about organ donation and organ transplantation. They want to know the health status of recipients after the operation. Besides, the personal privacy protection of donors and their families should be strengthened. Most of the interviewees in this study came from rural areas of China. The education level of rural residents is relatively low. So it is difficult for them to obtain information. Limited knowledge about organ donation leads to greater public pressure after organ donation. Thus, The government and the Red Cross Society of China should strengthen the propaganda of organ donation in economically underdeveloped areas. Measures need to be taken to improve the correct understanding and recognition of organ donation by citizens.

In terms of material needs, Financial assistance can be provided according to the organ donor familie's economic conditions (39). A study showed that the donor families had a large demand for material support, including medical needs (medical expenses reduction and exemption, organ transplantation priority treatment), social assistance (increasing endowment insurance, education fund and minimum living allowance, etc.,), and employment opportunities.

### Analysis of Social Support of Organ Donor Families

When it comes to organ donation, individuals should be encouraged to seek the help of family as well as friends, and they should be aware of the need for social support from family and friends during and after the decision (40). Anker AE pointed out that organ procurement coordinators (OPCs) identified six forms of emotional support and eight forms of instrumental support, with greater use of instrumental support strategies (41). The results showed that the family members of organ donors have received basic objective support, including direct material assistance, the transmission of organ donation-related information, medical services provided by medical institutions, and practical help. However, the sources of objective support for the donor families are limited. The main sources of financial support and practical help should come from spouses and relatives, while the support outside the family remained less. The subjective support of family members of organ donors is mainly emotional support and communication support. When seeking spiritual comfort and daily communication, family members tend to have personal relationships such as with relatives. Husband and wife and parents play an important supporting role. Stouder (42) has found that family support to be the most helpful in healing their grief, followed by friend's support, religious and cultural beliefs. Most of the family members talk to family members and get support from their immediate family members. This showed that the organ donor families have fewer approaches for psychological disclosure and their willingness to ask for help is not strong enough. It is found that the lower the utilization of social support, the lower the frequency of interaction with members will be. These results suggested that the utilization of social support from the family members of organ donors was low, and they did not take any initiative to seek support from the existing resources, leading to limited social support provided by others. Yang (39) has pointed out that it is their responsibility and obligation of the city and society to help the family after organ donation.

### Analysis of Influencing Factors of Social Support

Social support is closely related to coping styles. The more social support the individuals get, the more they tend to adopt positive coping styles. But the less social support they receive, the more likely they adopt negative coping styles such as evasion. As an intermediary mechanism of mental health and stress response, coping style plays an important role in the physical and mental health of the individual (43). Positive coping style showed a correlation with mental health, while negative coping style showed no correlation (44). In this study, the family members with positive coping styles obtained more social support than those with negative coping styles. Accumulated evidence suggests that social support is influenced by genetic and environmental factors (45). Except for the support from relatives for males, genetic factors cause variations in all dimensions of social support. Shared environmental factors influence relative support and relative problems in both sexes (46). Tess Thompson has found that neighborhood-socioeconomic deprivation and neighborhood-level social support affected the individual-level perception of social support indirectly through individual-level predictors in breast cancer patients, and to a lesser extent, controls (22). L M Sagrestano suggested that marital status is the most important predictor of support from a baby's father, whereas support from friends and family is more complex, and is associated with ethnicity, socioeconomic status, age, parity, and marital (47). The influencing factors of social support for rural children in China include supports from parents, teachers, peers, schools and social organizations (48). Kim et al. (49) found that the level of social participation showed the largest effect on a social support network. The other policy areas also showed positive significant influences to a social support network in the order of cultural and welfare policy, walking and local living environment, and local safety. Study on the correlation among resilience, social support and coping style of caregivers of family members in stroke patients showed that positive coping style can help family members to use more social support, which can increase the utilization of social support (50). The families of donors with positive coping styles will actively seek for help, actively express their personal needs, and use existing resources, such as family members, friends, coordinators, government and Red Cross organizations to seek support.

### Limitations

The current study had potential limitations. First, the sample size of this study is limited. The family members of organ donors are a special group of people. Being surveyed means that they would reminisce about the death of their families and experience the pain of losing their loved ones again, especially for respondents who participated in the semi-structured interview. Therefore, we use a combination of qualitative and quantitative methods to minimize the bias caused by the limited sample size.

## Conclusion

The level of social support and the utilization degree of social support among organ donor families is generally low. Organ donor families strongly hope to be understood by the public. Special organ donor families, such as the lost-one-child families, showed stronger social support needs, including material support needs and emotional support needs. Donor families have a great demand for material support, including medical needs, social assistance and employment opportunities. The coping style of organ donor families has an impact on the overall level of social support. Compared with a negative coping style, the family members of organ donors who adopt a positive coping style acquire more social support. Many families were found not having access to sufficient support in their networks, the need for government or Red Cross assistance was highlighted.

## Data Availability Statement

The raw data would be made available by the corresponding author on a reasonable request.

## Ethics Statement

The studies involving human participants were reviewed and approved by the Institutional Review Board of the Third Xiangya Hospital, Central South University (No.2016-S257). The patients/participants provided their written informed consent to participate in this study.

## Author Contributions

AJL participated in research design and data analysis. HYH participated in data collecting, data analysis, and paper writing. ZHX and XTD collected data and did the literature search. WZX participated in research design, data analysis, and paper writing. All authors approved the final submission.

## Funding

This research was supported by the Social Science Foundation of Hunan Province (16YBQ074) and the State Key Program of National Social Science of China (17AZD037).

## Conflict of Interest

The authors declare that the research was conducted in the absence of any commercial or financial relationships that could be construed as a potential conflict of interest.

## Publisher's Note

All claims expressed in this article are solely those of the authors and do not necessarily represent those of their affiliated organizations, or those of the publisher, the editors and the reviewers. Any product that may be evaluated in this article, or claim that may be made by its manufacturer, is not guaranteed or endorsed by the publisher.
